# Defining data librarianship: a survey of competencies, skills, and training

**DOI:** 10.5195/jmla.2018.306

**Published:** 2018-07-01

**Authors:** Lisa Federer

**Affiliations:** NIH Library, National Institutes of Health, Bethesda, MD

## Abstract

**Objectives:**

Many librarians are taking on new roles in research data services. However, the emerging field of data librarianship, including specific roles and competencies, has not been clearly established. This study aims to better define data librarianship by exploring the skills and knowledge that data librarians utilize and the training that they need to succeed.

**Methods:**

Librarians who do data-related work were surveyed about their work and educational backgrounds and asked to rate the relevance of a set of data-related skills and knowledge to their work.

**Results:**

Respondents considered a broad range of skills and knowledge important to their work, especially “soft skills” and personal characteristics, like communication skills and the ability to develop relationships with researchers. Traditional library skills like cataloging and collection development were considered less important. A cluster analysis of the responses revealed two types of data librarians: data generalists, who tend to provide data services across a variety of fields, and subject specialists, who tend to provide more specialized services to a distinct discipline.

**Discussion:**

The findings of this study suggest that data librarians provide a broad range of services to their users and, therefore, need a variety of skills and expertise. Libraries hiring a data librarian may wish to consider whether their communities will be best served by a data generalist or a subject specialist and write their job postings accordingly. These findings also have implications for library schools, which could consider adjusting their curricula to better prepare their students for data librarian roles.

## INTRODUCTION

As research becomes more data-intensive and researchers face new challenges in managing and sharing research data, libraries have begun to offer a variety of data support services, including instruction and training [1–4], data management planning guidance [5–7], data stewardship and curation [8–10], and data visualization [11–13]. Research over the last few years indicates a growth in library data services. While a 2012 study found only a “minority of US and Canadian academic libraries” provided some sort of data services [14], later research suggests that data services have become more common in libraries [15].

With data services playing an increasingly significant role in libraries’ offerings, some libraries have tasked liaison librarians with providing data services to their groups as part of their broader charges, while others have hired librarians and information specialists dedicated full-time to providing data services [16]. However, previous studies have suggested a lack of consensus around the definition of the data librarian role and the skills, education, and competencies required for such positions [15]. Competencies and skills have been developed for many other types of specialist information professionals. These documents help inform hiring decisions, continuing education, and curriculum development to ensure a workforce that is prepared to meet the information needs of the users they serve [17–19]. In the absence of an understanding in the profession of the data librarian role, library policy and strategic planning might not accurately reflect the experience of “frontline” data librarians [20], and librarians may even feel uncertain about exactly what “research data services” entails [21]. This uncertainty may also extend to iSchools, whose curriculum may be inadequate to prepare students to later fill data librarian positions [22].

This study aims to better define the skills, knowledge, and competencies that are essential to the data librarian role by surveying information professionals who self-identify as working in data librarian roles in order to explore the skills and knowledge that they consider most important to their work. In addition to identifying the types of skills and competencies that data librarians use in their work, this study also helps to more clearly define the profession of science data librarianship through an understanding of the characteristics of individuals who work in these roles. A better understanding of key competencies for science data librarians could also help create a workforce that is more prepared to take on such roles by informing library school curricula and continuing education offerings.

## METHODS

This study gathered information about skills and competencies from information professionals who spend a significant portion of their work providing data services. The survey instrument and recruitment materials did not specify the exact definition of “a significant portion of their work” in order to encourage respondents to complete the survey even if they were not engaged full-time in data-related work. Because the activities and information needs of researchers in the arts, humanities, and social sciences differed substantively from those of researchers in the sciences [23], the data services that information professionals provided to these groups likely differed from those provided to researchers working in the sciences. Therefore, this study focused on information specialists who provided services in biomedicine and the sciences, including those who provided services in these fields in addition to nonscience fields.

### Survey instrument

Data were collected using a survey instrument (supplemental Appendix A) consisting of questions about respondents’ educational and employment background and their perceptions of the importance of various skills and expertise to their current positions. The taxonomy of skills and expertise (supplemental Appendix B) was created by building upon existing lists of library skills and expertise to facilitate comparison of the present study to previous research [24–26], with the addition of data-related skills and expertise. Respondents rated the importance of these skills and expertise on a five-point Likert scale [27]. The survey instrument was developed in SurveyMonkey and distributed to four information professionals from the target population for pilot testing. Comments were collected from the pilot testers, and the instrument was revised accordingly. The final survey instrument was reviewed by the National Institutes of Health Office of Human Subjects Research Protections and determined to be excluded from institutional review board (IRB) review.

### Participant recruitment

Participants were invited to respond to the survey through several means. First, the survey was distributed to the email lists of the Medical Library Association (MLA) Medical Informatics Section and Data Special Interest Group, as well as the email list for Datacure, an informal group consisting of data specialists who work in libraries. In addition, the survey was sent to the email list of the Association of Academic Health Sciences Libraries, which primarily consists of library directors and managers, with the request to forward the announcement to data specialists working in their libraries. Finally, the survey was announced on Twitter and tagged with #medlibs, #datalibs, and #meddatalibs, which are commonly used to designate tweets relevant to medical librarians and data librarians. Because the survey was distributed through multiple media and because the proportion of recipients who were actually eligible to participate in the survey is unknown, the exact number of eligible individuals who received the announcement, and therefore the response rate, cannot be determined.

### Data analysis

At the end of the two-week survey period, responses were downloaded for analysis. Quantitative analysis was conducted using a variety of packages in R and RStudio, and visualizations were created using the ggplot2 package. Open-ended responses were analyzed manually. The full code for this analysis and the de-identified dataset are available on Open Science Framework.

In addition to basic statistical summary, cluster analysis was performed to detect subgroups within the sample [28]. The clustering algorithm finds respondents who are similar to each other based on their responses to the survey. Individuals within a group, or cluster, can be thought to be more similar to each other than to individuals who do not fall into that cluster. This type of analysis allows identification of latent classes in the data or groups of similar individuals, even if the researcher did not know in advance which groups would exist. Though a clustering algorithm can identify groups of similar individuals, it cannot identify the meaning or identity of the group. Human interpretation of the clusters must be done to determine what significance exists to the groups that were identified by the algorithm. For example, a clustering algorithm might identify two distinct groups of individuals in the dataset. The researcher would then review the characteristics of individuals in each of the two groups to determine what makes individuals in a group similar to each other and different from others and apply descriptive labels to these two groups.

In this study, similarity was calculated using Gower distance, which is suitable for noncontinuous and mixed variables [29], implemented in R using the cluster package [30]. Because nominal variables are not suitable for distance-based modeling, Likert scale data were one-hot encoded to binary variables [31]. After distances were calculated, clustering was conducted using the partitioning around medoids (PAM) algorithm [32]. Models were fit for between 2 and 10 clusters, and the 2-cluster model was determined to be the best fit, based on silhouette width [33]. Finally, hierarchical clustering analysis was performed using complete linkage to identify members of each of the 2 groups [34]. Statistical significance between the 2 groups was tested using Student’s 2-sample *t*-tests at a 95% confidence interval.

## RESULTS

A total of ninety participants responded to the survey during a two-week period in April 2017; eight surveys were discarded because the respondents had not completed enough responses, leaving eighty-two responses for the analysis.

### Participant characteristics

#### Job titles

Respondents who reported their job titles (n=81) had a variety of different titles; the only titles reported by more than 1 respondent were “data services librarian” (n=5) and “librarian” (n=2). While the other respondents’ job titles varied, some common words or phrases in titles included: “librarian” (n=47); “data services” (n=13); “research data” (n=11); “informationist” (n=6); “data management” (n=5); “manager” (n=5); and “director” (n=5).

#### Disciplinary support

More than two-thirds of respondents (n=55) supported more than 1 academic discipline, with a mean of 2.8 disciplines supported. The most common combinations of disciplines supported were biomedical or health sciences and life science (n=36) and engineering or computer science and physical sciences (n=28). Distributions of respondents supporting disciplines were: biomedical and/or health sciences: 82% (n=67); life sciences: 52% (n=43); engineering and/or computer science: 40% (n=33); social sciences: 38% (n=31); physical sciences: 38% (n=31); and mathematics and/or statistics: 30% (n=25). Seven respondents (9%) used the write-in “other” option to indicate that they also provided support to arts and/or humanities in addition to one of the scientific fields above.

#### Data versus non-data work

Respondents were asked to estimate the percentage of time that they spent working on data-related work versus other, non-data-related work. Of the respondents who provided an answer (n=78), only 5% (n=4) reported spending all of their time on data-related work. The majority of respondents (n=48, 59%) reported spending at least 50% of their time on data-related work, with a mean of 55% of time spent on data-related work. The distribution of time spent on data-related work was bimodal, with 11 respondents indicating that they spent 25% of their time on data-related work, and 12 respondents indicating that they spent 75% of their time on data-related work.

#### Experience in the field

Respondents reported the number of years that they had worked in their current positions, with times ranging between 0 and 17 years (mean=3.95 years). The most common length of time in the current positions was 1 year, with 25 respondents (31%). Respondents also indicated the number of years that they had worked in librarianship in total, with times ranging between 0 and 37 years (mean=9.99 years). The most common length of time in the field was between 4 and 6 years, with 30 respondents (38%) falling in this range.

Respondents’ number of years of experience at the time of starting their current positions was calculated by subtracting years in their current positions from their years in librarianship in total. Results ranged from 0 to 31 years (mean=6.04). The most common response was 0 years of experience at the time of starting the current position. Nearly 25% of respondents (n=20) indicated that their years in their current position were equal to their years in librarianship total. In other words, their current jobs could be assumed to be their first jobs in librarianship.

#### Educational experience

Respondents indicated their educational experience by selecting all applicable degrees and other educational opportunities, such as certificates and non-degree training, that they had completed. The majority of respondents had completed an American Library Association (ALA)–accredited master’s degree in library science or library and information science (MLS or MLIS), while other types of specialized education were less common. Most respondents (75%, n=62) reported completing more than 1 educational opportunity, with a mean of 2.23 completed educational opportunities. Distributions of respondents with various degrees were as follows: ALA-accredited master’s degree: 83% (n=68); undergraduate science degree: 39% (n=32); other nondegree, non-certificate training in data, science, or specialized librarianship: 38% (n=31); science master’s degree: 23% (n=19); doctorate (PhD) (any discipline): 17% (n=14); other non-ALA, nonscience master’s degree: 12% (n=10); and specialized librarianship certification (such as data or medical library certification): 11% (n=9).

### Importance of skills, knowledge, and competencies

**Figure 1:**
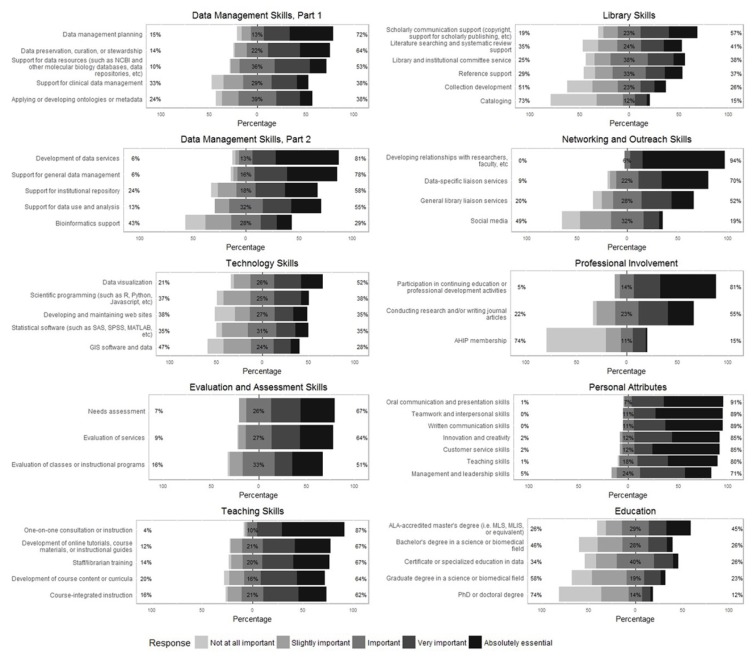
Overall ratings for each item, by category* * * The “Data Management” category is divided into two charts for ease of viewing due to the number of items in this category.

A taxonomy consisting of forty-seven items divided into nine categories (supplemental Appendix B) was created to quantify the types of skills, knowledge, and characteristics that data librarians consider most important to their work. Respondents were asked to rate the importance of each item to their work on a five-point Likert scale (“Not at all important,” “Important,” “Slightly important, “Very important,” and “Absolutely essential” [27], with an option for “Don’t know or N/A”). Figure 1 shows the breakdown of responses for each individual item in each of the nine categories.

“Personal Attributes” was the most highly rated category overall, with at least 70% of respondents ranking every item in that category “Very important” or higher. “Library Skills” was the lowest rated category, with about 40% of respondents or fewer ranking all but 1 item in that category “Very important” or higher. The top 5 items overall were: “Developing relationships with researchers, faculty, etc.”; “Oral communication and presentation skills”; “Teamwork and interpersonal skills”; “Written communication skills”; and “One-on-one consultation or instruction.” The bottom 5 items overall were: “PhD or doctoral degree”; “Academy of Health Information Professionals (AHIP) membership”; “Cataloging”; “Graduate degree in a science or biomedical field”; and “Collection development.”

### Cluster analysis

Using a clustering algorithm, 2 groups were identified in this data set. The 2 groups were analyzed and compared to determine their characteristics. One group, accounting for 60% of the sample (n=57) could be described as “subject specialists,” who tended to focus on a smaller number of disciplines and considered a smaller number of tasks to be important to their work. The other group, accounting for 30% of the sample (n=25) could be described as “data generalists,” who worked more broadly across disciplines and tended to rate a larger number of tasks as more highly important to their work. More data generalists reported spending most of their time on data-related work (80%) than did subject specialists (47%). Table 1 provides an overview of some of the differences between subject specialists and data generalists.

**Table 1:** Differences between respondents in subject specialists and data generalists clusters

	Subject specialists	Data generalists	*p*-value
Mean number of disciplines served	2.12	4.36	<0.001*
Mean degrees or certificates held	2.07	2.60	0.01*
Mean years in current position	4.89	3.2	0.13
Mean time in profession overall	10.25	10.04	0.92
Mean percent of time spent working on data-related tasks	47.27	73.2	<0.001*
Mean number of tasks rated absolutely essential	11.42	19.28	<0.001*

* *p*-values marked with * indicate a statistically significant difference between the 2 groups.

Considering the ratings of each group, data generalists tended to rate items as more important than the subject specialists, with a few exceptions. The items that subject specialists rated as more important than the data generalists reflected the more specialized areas that these respondents likely supported. For example, subject specialists rated “Bioinformatics support,” “Support for clinical data management,” and “Support for data resources (such as National Center for Biotechnology Information [NCBI] and other molecular biology databases, data repositories, etc.)” as more important overall than did data generalists. On the other hand, data generalists tended to rate skills related to data support in general—such as “Data management planning,” “Support for general data management,” and “Development of data services”—more highly than subject specialists did.

### Qualitative comments

In addition to the quantitative portion of the survey, respondents were invited to share other thoughts on skills and competencies for data librarianship, and thirty respondents provided written feedback. Most of these comments addressed training for data librarians, though many respondents differed in their opinions about which types of education were most important. A few respondents felt that an ALA-accredited master’s degree was crucial to success in data librarianship because such training provided a useful foundation in information retrieval, information-seeking behavior, scholarly communication, and other related knowledge. However, others disagreed on the relevance of the MLIS/MLS degree. One respondent noted, “I only rated the MLS as important because it’s still a credential that gets you in the door to working in a data-related position in a library. Very little of the MLS coursework I completed has been helpful in my current role.”

Many comments emphasized the importance of subject matter knowledge, whether that knowledge came in the form of formal scientific training, hands-on research experience, or even on-the-job learning. These respondents felt that a deep understanding of how and why research data are collected, organized, and analyzed as well as a familiarity with the scientific method and analytical processes were essential to the data librarian role. One respondent also commented that “our researchers have shown a strong bias towards working with ‘one of their own,’” suggesting that research experience or academic credentials might help bolster a data librarian’s credibility with users.

Just as the responses to the quantitative portion of the survey tended to emphasize the value of personal attributes over the technical skills, many comments highlighted that individual characteristics could be crucial to success. Respondents mentioned the importance of such traits as curiosity and the desire to learn, the ability to think “outside the box,” and a willingness to try new things.

## DISCUSSION

The findings of this study suggest that data librarians are a heterogeneous community of information professionals from varied educational and professional backgrounds, conducting many different types of work. As might be expected for an emerging profession, opinions differ among data librarians about the specific types of expertise that are important, and the types of work that different data librarians perform may be widely divergent. The existence of two groups of similar professionals, described here as subject specialists and data generalists, suggests that data librarianship may not be a single role but rather one that allows professionals to focus on areas related to their own interests or their users’ needs.

### Implications of study findings

These findings have implications for a number of a stakeholders, including libraries seeking to hire data librarians, educational institutions and the future data librarians they train, and data librarians themselves.

#### Implications for libraries

Libraries and institutions that aim to hire a data librarian may wish to consider whether their user population’s needs can best be met by a data generalist or a subject specialist data librarian. Data generalists, with a broad knowledge of how data are used across several subject areas or skills, may be well suited to work in academic settings where they will have opportunities to engage with students, faculty, and researchers in a variety of disciplines. On the other hand, subject specialist who have deeply developed a few select skills and cultivated expertise working with specific user groups may be a good fit for an institution where they can focus more specifically on the type of users who can benefit from their expertise.

Whatever type of data librarian institutions seek to hire, they may wish to carefully reflect on the actual skills and knowledge that will be necessary for individuals to be successful in the position. Many libraries have never had a dedicated, specialized data librarian before, so they may have little to draw on in terms of clearly defining the scope of a new data librarian job and may, therefore, have unclear expectations of the skills and knowledge that the successful job candidate should have. While this study shows that most data librarians consider about ten skills to be “absolutely essential” to their work, the content analysis portion of this study found that most job ads required about twenty skills. Rather than take a “kitchen sink” approach to job ads that includes a broad range of skills [35], institutions seeking to hire a data librarian can, in fact, identify candidates who are a better fit by reflecting on what the specific needs of the institution are as well as what types of skills professional data librarians consider most essential, as identified in research like the present study.

Once data librarians are hired, institutional support can help ensure that they are successful in their work. As several respondents noted, ongoing training is essential to ensure that data librarians remain up-to-date in a rapidly evolving field, so institutions may consider providing time and funding for data librarians to pursue such training. Because data librarianship can involve extensive outreach, including to high-level stakeholders at the institution, data librarians may also benefit from the support of their library leadership in forming these relationships. Given the emerging nature of the field, data librarians are often in the position of implementing new and previously untested services, some of which may end up being unsuccessful. Data librarians may be more effective in moving the profession forward in institutions where they feel that they are supported by their leadership and that they have the freedom to experiment and innovate, even if some of their projects ultimately fail.

#### Implications for educational institutions and future data librarians

The findings of this study also have implications for library schools and other institutions that are training the next generation of information professionals. Many respondents in this sample were ambivalent about the usefulness of their educational backgrounds to their current work, suggesting that new curricula may be needed to enable data librarians to adequately respond to the evolving needs of research communities. As the profession continues to evolve and new roles emerge, studies like this one can help identify the competencies that need to be incorporated into curricula for librarians and information professionals.

The emphasis on “soft skills” and personality traits may be encouraging to librarians who wish to transition into more data-focused roles, even if they do not have a highly technical or scientific background. Respondents in this study seemed to overwhelmingly agree that traits like oral communication skills and the ability to cultivate relationships with users were more important than highly technical skills like scientific programming or data visualization. Together with the finding that most respondents considered participation in continuing education important, these results suggest that librarians who are willing to learn more about research and the scientific process can be successful in data librarian positions.

#### Implications for data librarians

As has been discussed, what it really means to be a “data librarian” has not yet been clearly defined. This study has demonstrated that data librarians differ from one another in their professional expertise, the work they do, and even in characteristics as fundamental as their job titles. One respondent in this survey commented, “I feel less and less like a ‘librarian’ and more and more like...something else,” reflecting some of the uncertainty that comes with working in an emerging role.

While the actual practice of data librarianship can entail many different roles and skills, data librarians may benefit from the opportunity to engage with their peers through professional organizations and scholarly communication. Though no overarching professional organization exists for data librarians, special interest groups and other networking opportunities exist in several larger organizations. For example, MLA’s recently formed Data Special Interest Group provides an opportunity for data librarians to communicate with and learn from each other and creates a structure within which members can work to develop training on data-related topics for MLA members. Beyond medical librarianship, the Datacure email group allows data librarians working in a variety of roles and fields to communicate with each other. A recent article also provides suggestions for online communities and other resources for learning more about research data services [36]. These sorts of groups, both formal and informal, allow data librarians to explore challenges and opportunities as well as work together to help define the data librarian role.

### Limitations

More than 80% of the respondents in this sample reported supporting biomedical or health sciences. Because this survey was announced mostly through channels that reach medical librarians, this population was likely overrepresented in this sample. Therefore, the extent to which these results can be generalized to librarian data support in other disciplines may be limited. In addition, this survey utilized closed-ended questions to ask science data librarians about the importance only of the predefined set of skills included in the taxonomy. Though efforts were made to ensure that the taxonomy was complete and theoretically sound, additional skills that were not included in this taxonomy might also be relevant to science data librarianship.

### Conclusions and directions for future work

Future research that aims to reach other disciplinary populations could be useful in further expanding the knowledge of data librarianship beyond this article’s focus on biomedical and health sciences librarians. For example, many libraries are engaged in providing services to digital humanities researchers in ways that may differ from the types of data work described here [37]. Though data librarianship jobs are becoming more common, this field is still nascent, comprising a broad community of librarians with diverse job titles, backgrounds, and professional responsibilities. The field may remain similarly diffuse in the long-term, but future research could help determine whether the data librarian role will coalesce around a more defined professional identity.

Finally, support for researchers’ data needs is a moving target, with constantly evolving technologies and a quickly shifting policy landscape. Data librarians should keep their fingers on the pulse of their institutions’ needs to ensure that their skills, knowledge, and competencies remain relevant and up to date. Library schools and professional organizations should similarly stay up to date on trends in this rapidly evolving field to ensure that their curricula and continuing education programs are suitable to prepare information professionals to take on new data librarian roles.

## SUPPLEMENTAL FILES

Appendix A Data librarian skills and competencies survey

Appendix B Taxonomy of skills and expertise for data librarians
