# Supplementary material for: Defining data librarianship: a survey of competencies, skills, and training
Source: J Med Libr Assoc. 2018 Jul 1;106(3):294–303. doi: 10.5195/jmla.2018.306 (PMC6013124; doi:10.5195/jmla.2018.306)
Supplement: Appendix A [file jmla-106-294-s001.pdf]

JMLA Journal of the Medical Library Association

SC

Supplemental content to
J Med Libr Assoc. Jul;106(3):dx.doi.org/10.5195/jmla.2018.306
www.jmla.mlanet.org
© Federerer 2018

Defining data librarianship: a survey of competencies, skills, and training

Lisa Federer, MLIS, MA, AHIP

APPENDIX A

Data librarian skills and competencies survey

You are being invited to participate in a research study titled "'Databrarians' and Beyond: Competencies, Education, and Requirements for an Emerging Role." This study is being conducted by Lisa Federer of the National Institutes of Health (NIH) Library. The purpose of this research study is to gain a better understanding of skills and competencies of librarians and information professionals who perform data-related work.

You are eligible to participate in this survey if:

- a significant portion of your work is related to data services of some sort, and
- you work in a biomedical or science library, or work in a general academic library that serves biomedicine or sciences.

This survey will ask about your academic and professional background and your opinions on what skills and competencies are most important for data specialists in libraries. The survey should take you approximately 5 minutes to complete.

Your participation in this study is completely voluntary, and you can withdraw at any time. You are free to skip any question that you choose.

The results of this study will be presented at the Medical Library Association 2017 Annual Meeting in Seattle, WA. Your responses will be combined with others and will remain anonymous. Responses will be collected until Friday, May 5, 2017.

If you have questions about this project or if you have a research-related problem, you may contact Lisa Federer at lisa.federer@nih.gov. This research has been determined excluded from institutional review board (IRB) review by the NIH Office of Human Subjects Research Protections.

By clicking "Next" below you are indicating that you are at least eighteen years old, have read and understood this consent form, and agree to participate in this research study.

1. What is your job title?

---

2. For how many years have you worked in your **current** position?

---

3. For how many years **total** have you worked in a library setting?

Supplemental content to
J Med Libr Assoc. Jul;106(3):dx.doi.org/10.5195/jmla.2018.306
www.jmla.mlanet.org
© Federer 2018

4. Select the scientific area(s) you support in your data-related work.
- o Biomedical and/or health sciences
- o Life sciences
- o Physical sciences
- o Mathematics and/or statistics
- o Engineering and/or computer science
- o Social sciences
- o Other (please specify)

____

5. In an average week, what percentage of your time do you spend on data-related work and other work? (The sum of your responses should add up to 100.)
Percent of time spent on data-related work ________________________
Percent of time spent on other work ________________________

6. Which of the following educational experience or training have you completed? Check all that apply.
- o American Library Association (ALA)–accredited master's degree
- o Science master's degree
- o Other non-ALA, non-science master's degree
- o Undergraduate science degree
- o PhD (any discipline)
- o Specialized librarianship certification (such as data or medical library certification)
- o Other non-degree, non-certificate training in data, science, or specialized librarianship

Importance of qualification, knowledge, skills, and personal attributes

The following skills, knowledge, and characteristics have been identified as potentially relevant to work in data librarianship. For each skill or characteristic, please rate how important you consider each to work in your current position. You may also rate skills or knowledge you do not currently possess; for example, if you do not currently have expertise in scientific programming, but you think having this skill would help you be more successful in your position, you might rate it as important.

SC
Supplemental content to
J Med Libr Assoc. Jul;106(3):dx.doi.org/10.5195/jmla.2018.306
www.jmla.mlanet.org
© Federerer 2018

1. Data management

	Not at all important	Slightly important	Important	Very important	Absolutely essential	Don't know or N/A
Data management planning	O	O	O	O	O	O
Data preservation, curation, or stewardship	O	O	O	O	O	O
Applying or developing ontologies or metadata	O	O	O	O	O	O
Support for data resources (such as National Center for Biotechnology Information [NCBI] and other molecular biology databases, data repositories, etc.)	O	O	O	O	O	O
Support for clinical data management	O	O	O	O	O	O
Support for general data management	O	O	O	O	O	O
Bioinformatics support	O	O	O	O	O	O
Support for data use and analysis	O	O	O	O	O	O
Support for institutional repository	O	O	O	O	O	O
Development of data services	O	O	O	O	O	O

2. Technology and information technology (IT)

	Not at all important	Slightly important	Important	Very important	Absolutely essential	Don't know or N/A
Data visualization	O	O	O	O	O	O
Scientific programming (such as R, Python, Javascript, etc.)	O	O	O	O	O	O
Statistical software (such as SAS, SPSS, MATLAB, etc.)	O	O	O	O	O	O
Developing and maintaining websites	O	O	O	O	O	O
GIS software and data	O	O	O	O	O	O

SC
Supplemental content to
J Med Libr Assoc. Jul;106(3):dx.doi.org/10.5195/jmla.2018.306
www.jmla.mlanet.org
© Federer 2018

3. Evaluation and assessment

	Not at all important	Slightly important	Important	Very important	Absolutely essential	Don't know or N/A
Evaluation of classes or instructional programs	O	O	O	O	O	O
Evaluation of services	O	O	O	O	O	O
Needs assessment	O	O	O	O	O	O

4. Teaching and instruction

	Not at all important	Slightly important	Important	Very important	Absolutely essential	Don't know or N/A
Course-integrated instruction	O	O	O	O	O	O
Development of course content or curricula	O	O	O	O	O	O
Development of online tutorials, course materials, or instructional guides	O	O	O	O	O	O
One-on-one consultation or instruction	O	O	O	O	O	O
Staff/librarian training	O	O	O	O	O	O

5. Library skills

	Not at all important	Slightly important	Important	Very important	Absolutely essential	Don't know or N/A
Collection development	O	O	O	O	O	O
Library and institutional committee service	O	O	O	O	O	O
Reference support	O	O	O	O	O	O
Literature searching and systematic review support	O	O	O	O	O	O
Scholarly communication support (copyright, support for scholarly publishing, etc.)	O	O	O	O	O	O
Cataloging	O	O	O	O	O	O

JMLA Journal of the Medical Library Association

SC
Supplemental content to
J Med Libr Assoc. Jul;106(3):dx.doi.org/10.5195/jmla.2018.306
www.jmla.mlanet.org
© Federer 2018

6. Marketing and outreach

	Not at all important	Slightly important	Important	Very important	Absolutely essential	Don't know or N/A
Data-specific liaison services	O	O	O	O	O	O
General library liaison services	O	O	O	O	O	O
Social media	O	O	O	O	O	O
Developing relationships with researchers, faculty, etc.	O	O	O	O	O	O

7. Professional involvement

	Not at all important	Slightly important	Important	Very important	Absolutely essential	Don't know or N/A
Academy of Health Information Professionals membership	O	O	O	O	O	O
Participation in continuing education or professional development activities	O	O	O	O	O	O
Conducting research and/or writing journal articles	O	O	O	O	O	O

8. Skills and personal attributes

	Not at all important	Slightly important	Important	Very important	Absolutely essential	Don't know or N/A
Customer service skills	O	O	O	O	O	O
Innovation and creativity	O	O	O	O	O	O
Oral communication and presentation skills	O	O	O	O	O	O
Written communication skills	O	O	O	O	O	O
Teaching skills	O	O	O	O	O	O
Teamwork and interpersonal skills	O	O	O	O	O	O
Management and leadership skills	O	O	O	O	O	O

Journal of the Medical Library Association
Supplemental content to
J Med Libr Assoc. Jul;106(3):dx.doi.org/10.5195/jmla.2018.306
www.jmla.mlanet.org
© Federer 2018

9. Educational background

	Not at all important	Slightly important	Important	Very important	Absolutely essential	Don't know or N/A
ALA-accredited master's degree (i.e. MLS, MLIS, or equivalent)	O	O	O	O	O	O
Certificate or specialized education in data	O	O	O	O	O	O
Bachelor's degree in a science or biomedical field	O	O	O	O	O	O
Graduate degree in a science or biomedical field	O	O	O	O	O	O
PhD or doctoral degree	O	O	O	O	O	O

1. What other skills not included in the list on the last page do you consider important to your work in your current position?

____

2. Please share any other additional thoughts or comments you have related to skills and competencies for data specialists.

____
