# Supplementary material for: Defining data librarianship: a survey of competencies, skills, and training
Source: J Med Libr Assoc. 2018 Jul 1;106(3):294–303. doi: 10.5195/jmla.2018.306 (PMC6013124; doi:10.5195/jmla.2018.306)
Supplement: Appendix B [file jmla-106-294-s002.pdf]

JMLA Journal of the Medical Library Association

SC

Supplemental content to
J Med Libr Assoc. Jul;106(3):dx.doi.org/10.5195/jmla.2018.306
www.jmla.mlanet.org
© Federer 2018

Defining data librarianship: a survey of competencies, skills, and training

Lisa Federer, MLIS, MA, AHIP

APPENDIX B

Taxonomy of skills and expertise for data librarians

1. Data management
    a. Data management planning
    b. Data preservation, curation, or stewardship
    c. Developing or applying ontologies and metadata
    d. Support for data resources (such as National Center for Biotechnology Information [NCBI] and other molecular biology databases, data repositories, etc.)
    e. Support for clinical data management
    f. Support for general data management
    g. Bioinformatics support
    h. Support for data use and analysis
    i. Support for institutional repository
    j. Development of data services

2. Technology and information technology (IT)
    a. Data visualization
    b. Scientific programming (such as R, Python, Javascript, etc.)
    c. Statistical software (such as SAS, SPSS, MATLAB, etc.)
    d. Developing and maintaining websites
    e. Geographic information system (GIS) software and data

3. Evaluation and assessment
    a. Evaluation of classes or instructional programs
    b. Evaluation of services
    c. Needs assessment

4. Teaching and instruction
    a. Course-integrated instruction
    b. Development of course content or curricula
    c. Development of online tutorials, course materials, or instructional guides
    d. One-on-one consultation or instruction
    e. Staff or librarian training

5. Marketing and outreach
    a. Data-specific liaison services
    b. General library liaison services
    c. Social media
    d. Developing relationships with researchers, faculty, etc.

SC
Supplemental content to
J Med Libr Assoc. Jul;106(3):dx.doi.org/10.5195/jmla.2018.306
www.jmla.mlanet.org
© Federerer 2018

6. Library skills
    a. Collection development
    b. Library and institutional committee service
    c. Reference support
    d. Literature searching and systematic review support
    e. Scholarly communication support (copyright, support for scholarly publishing, etc.)
    f. Cataloging

7. Professional involvement
    a. Academy of Health Information Professionals membership
    b. Participation in continuing education or professional development activities
    c. Conducting research and/or writing journal articles

8. Skills and personal attributes
    a. Customer service skills
    b. Innovation and creativity
    c. Oral communication and presentation skills
    d. Written communication skills
    e. Supervisory skills
    f. Teaching skills
    g. Teamwork and interpersonal skills
    h. Management and leadership skills

9. Education and training
    a. American Library Association (ALA)–accredited master's degree
    b. Science master's degree
    c. Other non-ALA, non-science master's degree
    d. Undergraduate science degree
    e. Doctorate (PhD)
    f. Specialized librarianship certification (such as data or medical library certification)
